# Proposed Changes to U.S. Policy on Potential Pandemic Pathogen Oversight and Implementation

**DOI:** 10.1128/mSphere.00990-19

**Published:** 2020-01-22

**Authors:** Thomas V. Inglesby, Marc Lipsitch

**Affiliations:** a Center for Health Security, Johns Hopkins Bloomberg School of Public Health, Baltimore, Maryland, USA; b Center for Communicable Disease Dynamics, Departments of Epidemiology and Immunology and Infectious Diseases, Harvard T.H. Chan School of Public Health, Boston, Massachusetts, USA

**Keywords:** biosafety, biosecurity, policy, potential pandemic pathogen, research regulation

## Abstract

We propose here changes to the U.S. government policy on potential pandemic pathogen (PPP) oversight and implementation, emphasizing transparency of the review process and the content of the review, publication of the review in advance, responsible publication of enhanced PPP research, high-level signoff on approvals of enhanced PPP experiments, and the need for a significant effort to establish a common international approach to enhanced PPP work. We advocate that the U.S. government recommend, and non-U.S. government funders and journals adopt, a set of best practices that would extend important considerations of biosafety and biosecurity to all work on enhanced potential pandemic pathogens regardless of funding source.

## Abstract (precis)

We propose here changes to the U.S. government policy on potential pandemic pathogen (PPP) oversight and implementation, emphasizing transparency of the review process and the content of the review, publication of the review in advance, responsible publication of enhanced PPP research, high-level signoff on approvals of enhanced PPP experiments, and the need for a significant effort to establish a common international approach to enhanced PPP work. We advocate that the U.S.

## COMMENTARY

In December 2017, the U.S. Department of Health and Human Services (HHS) published the *HHS Framework for Guiding Funding Decisions about Proposed Research Involving Enhanced Potential Pandemic Pathogens* (HHS P3CO Framework) (1). This framework was based on earlier guidance on this subject issued by the White House Office of Science and Technology Policy (OSTP) in January 2017 (2). The HHS framework defines a potential pandemic pathogen (PPP) as a pathogen that is both “likely highly transmissible and likely capable of wide and uncontrolled spread in human populations” and “likely highly virulent and likely to cause significant morbidity and mortality in humans.” In January 2019, it was reported (3) that HHS approved new enhanced PPP experiments, and this occurred without public notification or public description of the process related to their approval.

The OSTP guidance included a plan for evaluation of agency actions. It called for an OSTP assessment of the impact of the policy on research programs and institutions, of the impact on enhanced PPP research, and of “how to provide transparency, public engagement, and continued dialogue about enhanced PPP research” (2). In addition, in January 2020 there will be a meeting of the National Science Advisory Board for Biosecurity (NSABB), which will focus on balancing the issues of transparency and security when communicating about research involving pathogens with pandemic potential. Given the requirement for an OSTP assessment of the guidance and HHS Framework and the planned NSABB meeting, we have a number of recommendations regarding how the HHS Framework and the OSTP guidance should be amended so that “biosafety and biosecurity risks associated with undertaking such research [are] adequately considered and appropriately mitigated in order to safely realize the potential benefits” (1).

## MAKE THE HHS REVIEW OF ENHANCED PPP EXPERIMENTS TRANSPARENT

Currently, none of the HHS departmental review process for approving enhanced PPP experiments is public. This is inconsistent with the OSTP guidance which said: “To the maximum extent possible, agencies’ enhanced PPP review mechanisms should provide transparency to the public regarding funded projects involving the creation, transfer or use of enhanced PPPs” (2). To that end, the HHS review should make public who participates in the review, as well as the basis of the decision that the research is acceptable to fund, including the U.S. government’s (USG’s) calculation of the potential benefits and risks of the proposed enhanced PPP research.

The HHS P3CO Framework says that the following disciplines should be represented in the HHS review: “scientific research, biosafety, biosecurity, MCM development and availability, law, ethics, public health preparedness and response, biodefense, select agent regulations, and public health policy.” But there has been no public description of who has been part of these reviews. This is distinct from NIH reviews where review committee rosters are public. Public description of who has been represented in the review is important for public accountability to ensure the Framework is being followed appropriately. While full independence of reviewers as in the scientific grant review process may not be practical in this setting, and the guidance indicates that it should include “funding agency perspectives,” the review will gain credibility if the majority of the experts assembled for this work are free of institutional conflict of interest (e.g., employment by the funding agency or its parent or sister agencies), a goal most readily achieved by using experts from the academic or nonprofit sectors.

In addition, the approval of state public health authorities (or local designates, as appropriate) should be required for enhanced PPP experiments, as was required for the approval of the biosafety level 4 (BL4) lab in Boston, MA (4). That process reflected the fact that BL4 laboratories could pose local risks of infections to laboratory personnel and immediate contacts. Public health approval of enhanced PPP experiments would reflect that this research could pose a risk of a local epidemic, which could further expand to a global pandemic in case of failure of local control. For potential pandemic pathogens, local awareness and acceptance of biosafety risks are all the more pressing than with less transmissible pathogens often studied in BL4 labs, because of the global stakes when PPPs are involved.

Beyond the procedural points made so far, the substance of the deliberations should also be public. None of the details of the analysis related to the HHS decisions approving the 2019 enhanced PPP experiments has been publicly released. Without a publicly released analysis of these experiments, there is no basis for understanding the HHS decision that the research is acceptable. The USG should provide its official assessment of the potential benefits and risks of any PPP experiment that is approved. The USG has not published qualitative or quantitative benefit and risk assessments for the specific 2019 enhanced PPP research that it has already approved. These analyses should be publicly released now so that the scientific community and the public can understand HHS decision-making.

For any future proposed enhanced PPP research, this kind of risk assessment, including any dissenting views, should be published in advance of the provision of any funding of the experiments. It is recognized that this exceeds the level of transparency required for ordinary public funding reviews. No reviews are publicly released for a typical NIH grant—appropriately reflecting that the decision is a competition between different scientific uses of scarce funds and the risk of choosing one is simply the opportunity cost of not choosing another. The unprecedented risk posed by PPP research justifies a higher level of transparency, appropriately balancing public safety (rather than just the public purse) against the private interests of researchers in the confidentiality of their science. Where possible without compromising transparency of the decision, particular details of the proposed experiments may be omitted from public disclosures if revealing them would compromise the competitive position of the researchers, but the guiding principle should be that a concern for transparently guaranteeing public safety outweighs a concern for researchers’ trade secrets.

## PROVIDE THE PLAN FOR “RESPONSIBLE” PUBLICATION OF ENHANCED PPP RESEARCH

The HHS P3CO Framework states: “if funded, research that is reasonably anticipated to create, transfer or use an enhanced PPP may require additional risk mitigation strategies which may include, but are not limited to: …methodologies for responsible communication of results.” There is no definition or clarity in the Framework regarding methodologies of responsible communication of results. Given the appropriate requirement of funders like NIH for open publication of results, the results of NIH-funded PPP work will be available everywhere globally from that point forward. Sequence data would facilitate the reconstruction of the enhanced potential pandemic pathogen. It is impossible to control where such efforts to duplicate the work would take place. Moreover, journal requirements for resource sharing postpublication might require researchers to share enhanced PPPs or reagents to create them with parties whose possession of them would threaten security and/or safety. While HHS might have determined that the original enhanced PPP experiments were taking place at an institution capable of sufficient biosafety and biosecurity controls, they cannot know the context or biosafety or biosecurity conditions that other scientists will employ in efforts to reproduce the research or use the products thereof.

On the other hand, if the work is done in a classified setting (e.g., if supported by an agency, such as the Department of Defense, which funds classified research), other countries may be concerned that these experiments are secret and being done behind closed doors. For this reason, it is important that HHS explains now, before additional decisions are made regarding the approval and funding of this work, what its requirements will be regarding “responsible communication” of the results of this research.

When enhanced PPP work is performed with USG funding, special consideration should be given to policies on resource sharing and related issues, to prevent the sharing of enhanced PPPs or the reagents to create them if such sharing could itself create an unacceptable biosafety or biosecurity risk. In cases of research approved under the HHS P3CO Framework, the presumption should be against resource sharing, in contrast to ordinary science where the presumption (or even requirement) is in the other direction.

## ESTABLISH A COMMON INTERNATIONAL APPROACH TO ENHANCED PPP RESEARCH

The January 2017 OSTP P3CO guidance stated that “the US government should engage with other countries about policies concerning creation, transfer and use of enhanced PPP, encouraging the development of harmonized policy guidance” (2). However, to our knowledge there has been no robust attempt at international consensus building or harmonization since the publication of this policy. Given the high stakes involved and the leadership role that the United States has in the life sciences globally, the United States can and should take the lead internationally in establishing discussions with other science funding agencies and national academies on enhanced PPP issues. Without clear international outreach from the USG, countries can assume that since the United States is approving and funding this work, they too should be able to approve and fund this work. However, it is not in the interest of the United States or any country for countries to be funding this work without a very compelling rationale, rigorous reviews, and the highest possible biosafety and biosecurity standards in place.

If this work is going to be funded by the USG and other governments, it would be in the interest of all countries if this work was restricted to the smallest number of laboratories that have globally exceptional records of biosafety and biosecurity, experience with dangerous pathogens of the type under study, staff training, strong security procedures, and state-of-the-art-facilities that operate under an appropriate national policy framework that ensures the safety of the work.

## REQUIRE A HIGH-LEVEL OFFICIAL, SUCH AS THE NIH DIRECTOR OR HHS SECRETARY, TO APPROVE ENHANCED PPP RESEARCH

The *NIH Guidelines for Research involving Recombinant or Synthetic Nucleic Acid Molecules* states that “the deliberate transfer of a drug resistance trait to a microorganism when such resistance could compromise the ability to control the disease agent in humans, veterinary medicine, or agriculture” requires “Major Action,” which is the requirement for the signature of the NIH Director (5). Given that the potential consequences of enhanced PPP research are the initiation of an epidemic or pandemic that may not be able to be stopped with a vaccine or antiviral, it seems like this approval should similarly require the signature approval of the NIH Director or HHS Secretary. Currently, it is not clear at what level of government this approval is made.

## DEVELOP GUIDANCE FOR JOURNALS AND OTHER FUNDERS OF BIOMEDICAL RESEARCH TO EMBODY THE SPIRIT AND GOALS OF THE HHS P3CO FRAMEWORK

Best-practice guidance should be developed to encourage responsible actions by non-USG funders and by publishers of scientific journals. Such best practices should be institutionalized, for example, according to the precedent of NIH recombinant DNA guidelines, which apply to research at institutions receiving federal funding for recombinant DNA work and their collaborators, regardless of the direct source of the funds for the specific research in question (6). These best practices should include the following:

For funders:

1. Funders should establish a set of criteria for flagging research of potential concern for enhanced PPP work, ideally following the USG criteria.
2. Funders should establish policies and procedures for high-level review of research meeting such criteria, again mirroring to the extent possible the USG policies and procedures. This is consistent with OSTP guidance on this issue which called for consideration of extending P3CO policy guidance in ways that “would enable oversight of all relevant research activities, regardless of funding source” (2). A best practice would be to establish a transparent review committee with comparable disciplinary expertise to that laid out in the P3CO guidance for USG funding decisions; public disclosure prior to approval of the deliberations, decision, and reasoning of the review committee, including dissenting views; and approval by the top official of the funding body only upon a favorable finding by the review committee.

For publishers:

1. Any journal submission of enhanced PPP work, as defined by the P3CO guidance, regardless of funding source, should be considered for publication only upon submission of the transparent reporting of the funding source, USG or otherwise, of the reviews described above and in the P3CO guidance, including the identity of reviewers, their qualifications, the risk and benefit calculations, and dissenting views if present.
2. Enhanced PPP work as defined by the P3CO guidance should be peer reviewed by experts in biosafety and biosecurity along with scientific reviewers, regardless of funding source. These reviewers should be asked to flag any specific issues of biosafety and biosecurity raised by the publication of the work, as well as evaluating the adequacy of the risk-benefit assessment. Publication should be contingent on the acceptance of these reviewers that the publication would be acceptable from a biosafety and biosecurity perspective.
3. Journals should make exceptions to policies on reproducibility and resource sharing that normally apply to all published articles in the event that such sharing would create a concern of biosafety or biosecurity. For example, enhanced PPPs produced by a published article or the reagents to create them, sharing of which might normally be required by journal policies, might be exempted from such policies except in cases where the receiving party has a demonstrated need for them and a valid set of permissions from relevant authorities to work with them. Details of this policy would need further development.

## CONCLUSION

The HHS P3CO Framework was created to guide funding decisions for enhanced potential pandemic pathogens because it was recognized that such work posed biosecurity and biosafety risks at the population level that required special consideration and approaches. In order to properly address such risks, this framework and its implementation should become transparent, articulate its plan for responsible communication, robustly pursue international engagement and harmonization, require the signature approval of the HHS secretary or NIH Director for funding of enhanced PPP research, and develop guidance for nongovernmental funders and publishers of this work. These changes would substantially increase scientific and public understanding of this process and lower the risks associated with PPP research.
